# Functional epigenomics approach to identify methylated candidate tumour suppressor genes in renal cell carcinoma

**DOI:** 10.1038/sj.bjc.6604180

**Published:** 2008-01-15

**Authors:** M R Morris, D Gentle, M Abdulrahman, N Clarke, M Brown, T Kishida, M Yao, B T Teh, F Latif, E R Maher

**Affiliations:** 1 Cancer Research UK Renal Molecular Oncology Group, University of Birmingham, Birmingham B15 2TT, UK; 2 Department of Medical and Molecular Genetics, Department of Paediatrics and Child Health, University of Birmingham, Birmingham B15 2TT, UK; 3 Paterson Institute for Cancer Research, University of Manchester, Manchester, M20 4BX, UK; 4 Yokohama City University School of Medicine, Yokohama, Japan; 5 Laboratory of Cancer Genetics, Van Andel Research Institute, Grand Rapids, MI, USA

**Keywords:** renal cell carcinoma, methylation, epigenetics

## Abstract

Promoter region hypermethylation and transcriptional silencing is a frequent cause of tumour suppressor gene (TSG) inactivation in many human cancers. Previously, to identify candidate epigenetically inactivated TSGs in renal cell carcinoma (RCC), we monitored changes in gene expression in four RCC cell lines after treatment with the demethylating agent 5-azacytidine. This enabled us to identify *HAI-2*/*SPINT2* as a novel epigenetically inactivated candidate RCC TSG. To identify further candidate TSGs, we undertook bioinformatic and molecular genetic evaluation of a further 60 genes differentially expressed after demethylation. In addition to *HAI-2*/*SPINT2*, four genes (*PLAU*, *CDH1*, *IGFB3 and MT1G*) had previously been shown to undergo promoter methylation in RCC. After bioinformatic prioritisation, expression and/or methylation analysis of RCC cell lines±primary tumours was performed for 34 genes. *KRT19* and *CXCL16* were methylated in RCC cell lines and primary RCC; however, 22 genes were differentially expressed after demethylation but did not show primary tumour-specific methylation (methylated in normal tissue (*n*=1); methylated only in RCC cell lines (*n*=9) and not methylated in RCC cell lines (*n*=12)). Re-expression of *CXCL16* reduced growth of an RCC cell line *in vitro*. In a summary, a functional epigenomic analysis of four RCC cell lines using microarrays representing 11 000 human genes yielded both known and novel candidate TSGs epigenetically inactivated in RCC, suggesting that this is valid strategy for the identification of novel TSGs and biomarkers.

Renal cell carcinoma (RCC) is a heterogeneous disorder. Most (∼75%) of the tumours are classified as clear cell (conventional) and the next most frequent subtype is papillary RCC (∼15% of all cases) (Kovacs *et al*, 1997). The most frequent genetic event in the evolution of clear cell RCC is inactivation of the *VHL* tumour suppressor gene (TSG) (Latif *et al*, 1993; Foster *et al*, 1994; Herman *et al*, 1994; Clifford *et al*, 1998), but epigenetic inactivation of TSGs by methylation of CpG dinucleotides in the promoter region has also been implicated in the pathogenesis of RCC (Morrissey *et al*, 2001; Dallol *et al*, 2002; Morris *et al*, 2003). Tumour suppressor gene promoter methylation has been studied most extensively in colorectal cancer, but TSGs that are frequently methylated in human cancers (e.g. *p16^ink4a^*, *DAPK*, *NORE1A*, *MGMT*, *SDHB*, *RARB2* and *CDH13*) are infrequently methylated in RCC (Morris *et al*, 2003). This observation prompted us to speculate that, compared to other tumour types, a different repertoire of TSGs might undergo epigenetic inactivation in RCC. Therefore, to identify candidate novel epigenetically inactivated RCC TSGs, we performed a gene expression profiling of four RCC cell lines treated with the demethylated agent 5-azacytidine (5-AZA) (Morris *et al*, 2005). Analysis of genes differentially expressed before and after demethylation led us to identify *HAI-2*/*SPINT2* as a novel epigenetically inactivated RCC TSG (Morris *et al*, 2005). In this study, we report the further analysis of our functional epigenomic screen to identify genes relevant in development of RCC.

## MATERIALS AND METHODS

### Cell Lines, 5-AZA-dC treatment and microarray analysis

Full details of the gene expression microarray experiments on four RCC cell lines (KTCL26, SKRC39, SKRC45 and SKRC47) have been described previously (Morris *et al*, 2005). All RCC cell lines (KTCL26, RCC4, UMRC2, UMRC3, SKRC18, SKRC39, SKRC45, SKRC47, SKRC54, 786-0 and Caki-1) analysed in this study were routinely maintained in DMEM (Invitrogen, San Diego, CA, USA) supplemented with 10% FCS at 37°C, 5% CO_2_. The demethylating agent 5-AZA-dC (Sigma, Gillingham, UK) was freshly prepared in ddH_2_O and filter sterilised. Cell lines were plated in 75-cm^2^ flasks in DMEM supplemented with 10% FCS at differing densities, depending upon their replication factor, to ensure that both control and 5-AZA-dC-treated lines reached approximately 75% confluency at the point of RNA extraction. Twenty-four hours later, cells were treated with 5 *μ*M 5-AZA-dC. The medium was changed 24 h after treatment and then changed again after 72 h. RNA was prepared 5 days after treatment using RNABee (AMS Biotechnology, Oxon, UK).

### Patients and samples

DNA from a total of 127 primary RCCs and 6 non-cancer-related kidneys was analysed. Local ethics committees approved the collection of samples and informed consent was obtained from each patient.

### RT–PCR conditions

PCR cycling conditions consisted of an initial denaturing step of 95°C for 5 min, followed by 30 cycles of 95°C denaturing at 45 s, primer annealing at 55–60°C (gene-dependent) and product extension at 72°C for 45 s. Semiquantitative analysis of expression was performed using LabWorks software (Ultraviolet products, Upland, CA, USA). *GAPDH* primers were 5′TGAAGGTCGGAGTCAACGGATTTGGT3′ and 5′CATGTGGGCCATGAGGTCCACCAC3′ (other RT–PCR primers and conditions upon request).

### Bisulphite modification and methylation analysis

Bisulphite DNA sequencing was performed as described previously (Morris *et al*, 2003). Briefly, 0.5–1.0 *μ*g of genomic DNA was denatured in 0.3 M NaOH for 15 min at 37°C, and then unmethylated cytosine residues were sulphonated by incubation in 3.12 M sodium bisulphite (pH 5.0; Sigma)/5 mM hydroquinone (Sigma) in a thermocycler (Thermo Fisher Scientific, Waltham, MA, USA) for 20 cycles of 30 s at 99°C and 15 min at 50°C. The sulphonated DNA was recovered using the Wizard DNA cleanup system (Promega, Southampton, UK) in accordance with the manufacturer's instructions. The conversion reaction was completed by desulphonating in 0.3 M NaOH for 10 min at room temperature. The DNA was ethanol-precipitated and resuspended in water.

### Promoter methylation analysis

CpG islands were identified on the human genome browser and putative promoter regions were predicted by Promoter Inspector software (Genomatix, www.genomatix.de). Primer details are shown in Supplementary Table 1.

### Plasmid constructs and colony formation assay

The *CXCL16* expression construct was made by cloning the full-length human *CXCL16* coding region from the SKRC 18 kidney cell line, into the *Eco*R1–*Bam*HII sites of pCDNA3.1 vector (Invitrogen). Plasmid constructs were verified by sequencing. Six micrograms of empty vector or 6.8 *μ*g (equal molar amounts) of expression vector was transfected, by calcium phosphate method, into 5 × 10^5^ SKRC39 cells. Forty-eight hours after transfection, cells were seeded in a serial dilution and maintained in DMEM and 10% foetal bovine serum supplemented with 1 mg ml^−1^ G418 (Gibco, Invitrogen, Paisley, UK). Surviving colonies were stained with 0.4% crystal violet (Sigma) in 50% methanol, 21 days after initial seeding, and counted. Each transfection was carried out in triplicate. Additionally, replicate experiments were carried out to obtain further clones for expression analysis.

## RESULTS

### Identification and evaluation of differentially expressed genes after demethylation of RCC cell lines

A total of 56 genes (of 11 000 transcripts analysed), each with a 5′ CpG island, that demonstrated >5-fold increased expression in one cell line or >2-fold in multiple cell lines were identified as candidate epigenetically inactivated RCC TSGs. In addition to *SPINT2*, four further genes (*CDH1*, *PLAU*, *IGFB3* and *MT1G*) had previously been reported to undergo promoter region hypermethylation in RCC tumours (Yoshiura *et al*, 1995; Morris *et al*, 2003; Ibanez de Caceres *et al*, 2006). As we wished to identify novel genes, these were not analysed further. After bioinformatic evaluation (e.g. renal tissue specific expression patterns (array express (www.ebi.ac.uk/arrayexpress/), human genome browser (www.genome.ucsc.edu)), analysis of CpG islands for the presence of predicted promoter regions (www.genomatix.de) and consideration of likely role in tumorigenesis), 34 genes were selected for analysis of expression and/or CpG island methylation status in RCC cell lines±primary RCC tumours.

After analysis of expression pre- and post-treatment with 5-AZA in up to 11 RCC cell lines, nine genes (*ATF5*, *SLC1A4*, *ID3*, *STC2*, *DUSP6*, *SEMA3C*, *CD44*, *HMGA1* and *IRF7*) were excluded from further investigation as they were not commonly silenced in the pretreatment cell lines (data not shown).

### Promoter region methylation analysis of RCC cell lines and tumours

All genes analysed had a 5′ CpG island (as identified by the human genome browser (http://genome.ucsc.edu/)) and promoter regions were predicted by Genomatix promoter inspector software (http://www.genomatix.de/). The methylation status of 5′ CpG island promoter regions were analysed by direct sequencing of bisulphate-modified DNA of RCC cell lines and primary tumours for 25 genes. Methylation of 5′ CpG dinucleotides was rare (*ICAM1*, *IGSF4*, *EHM2*, *CLDN1*, *MUC1*, *SEMA5A*) or absent (*CTGF*, *PHD3*, *RRM2*, *PMAIP1*, *GPR39*, *MYL2*, *BAP1*, *SLC25A21*, *FBLN1*, *H2B*, *ECE1*, *FZD8*) for 18 genes. For *ICAM1*, *IGSF4*, *EHM2*, *CLDN1* and *MUC1*, no methylation was detected in 20 sporadic RCC tumours. These findings suggest that the alterations in expression after demethylation observed for these 18 genes were caused either by (a) methylation of other *in cis* regulatory regions or (b) that expression of these genes might be regulated by genes that were epigenetically inactivated.

**Figure 1:**
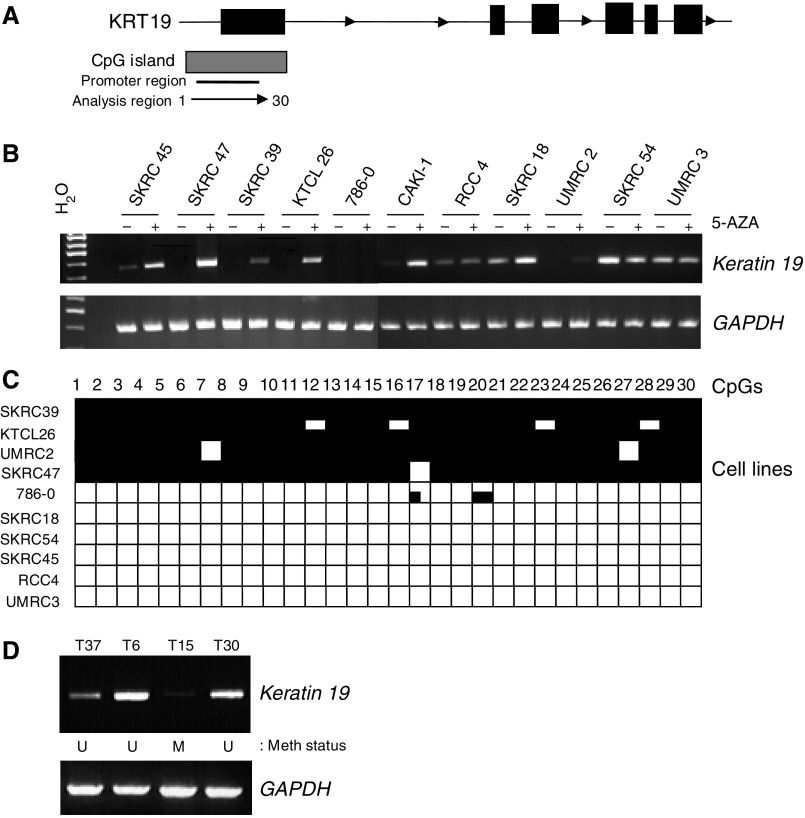
Tumour-specific *KRT19* promoter methylation. (**A**) Schematic of CpG island and predicted promoter region in relation to the *KRT19* gene. (**B**) RT–PCR analysis of *KTN19* shows silencing in five RCC cell lines. Expression is reactivated in four lines following treatment with 5-AZA. (**C**) Promoter region methylation analysis by direct sequencing indicates that methylation correlates with gene silencing. (**D**) Methylation correlated to expression in tumours; compare the methylation status of samples 15T and 30T with expression by RT–PCR. T, tumour, M, mehtylated promoter, U, unmethylated promoter.

Seven genes demonstrated promoter region hypermethylation concordant with gene expression in RCC cell lines (e.g. *KTN19*; Figures 1A–C). Four of the seven genes (*SST*, *PTGS1*, *ISG15*, *THY1*) were frequently methylated in RCC cell lines (Table 1) but were not methylated in primary RCC (*n*=20) and although *ENG* was frequently methylated in RCC cell lines and primary tumours (Table 1), methylation was also detected in adjacent normal tissues and in normal renal tissue from patients without cancer (*n*=6).

Two of the seven genes, *CXCL16* and *KTN19*, demonstrated frequent promoter region hypermethylation in RCC cell lines (40 and 62%, respectively) and in primary RCCs (42% (*n*=62) and 38% (*n*=66), respectively, but not in normal renal tissue from patients without cancer (*n*=6). For primary RCC with *KTN19* promoter methylation, methylation was rarely (14%, 3/22) detected in the adjacent normal tissue. In contrast, for tumours with CXCL16 methylation (but not for unmethylated tumours), methylation was also detected in the adjacent normal tissue.

We have expanded our previous analysis of *HAI-2*/*SPINT2* promoter methylation in RCC (38%) to incorporate all tumours analysed for *KTN19* and *CXCL16* promoter methylation. However, no significant correlation was detected between methylation at *SPINT2*, *KTN19* and *CXCL16* (*P*>0.1) (data not shown).

### RE-expression of *CXCL16* reduces the colony forming ability of kidney-derived cell lines

**Figure 2:**
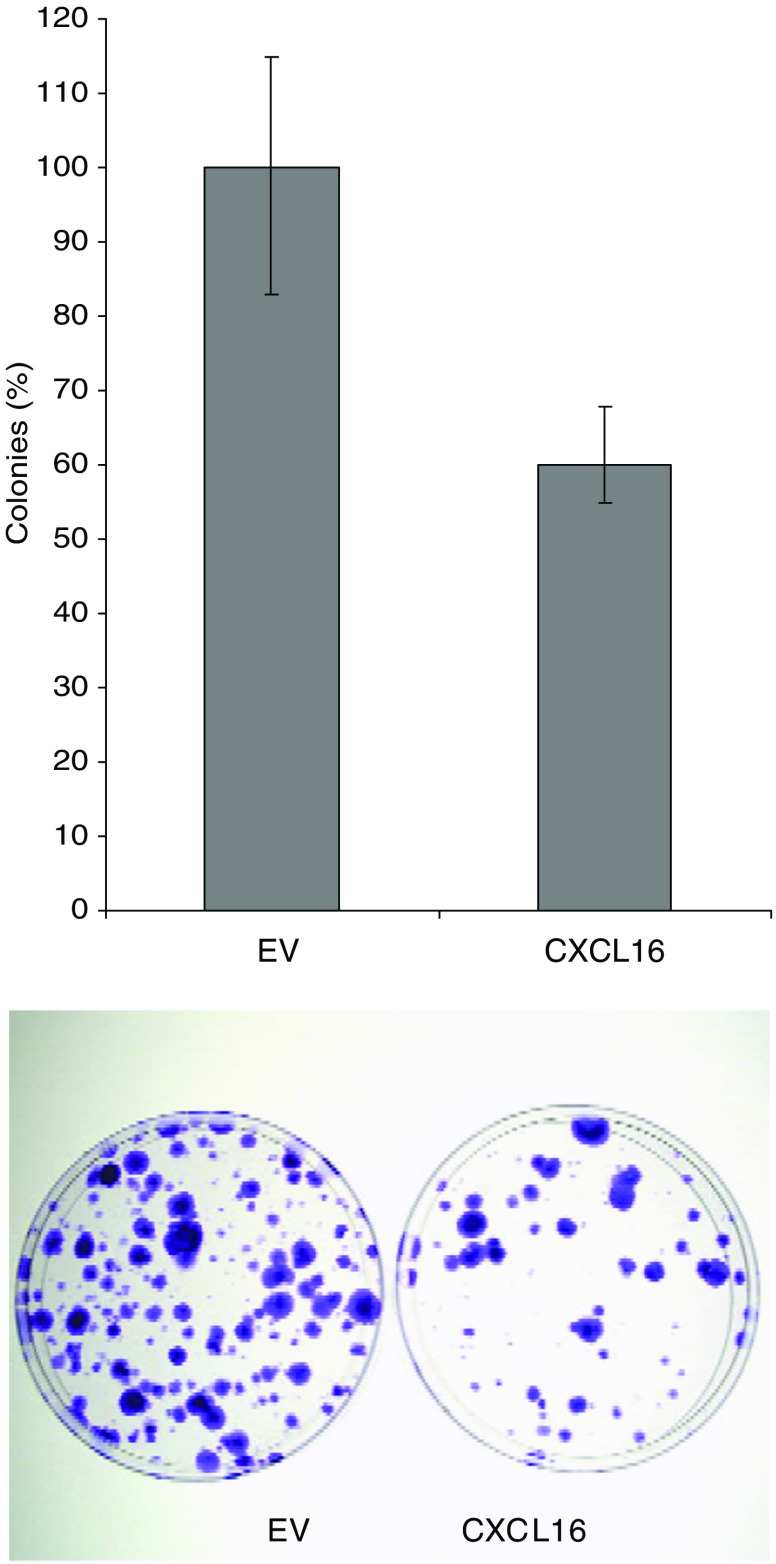
Re-expression of *CXCL16* in RCC cells results in growth suppression. Equal (molar) amounts of empty vector (EV) and pCDNA3.1-CXCL16 (*CXCL16*) were transfected into SKRC39 cells. Following antibiotic selection, surviving colonies were stained. Each experiment was performed in triplicate and the means of these used to produce the bar chart. The mean number of colonies counted in the EV plates was taken as 100%. There was a statistically significant reduction of colonies in each of the *CXCL16* transfectants (*P*=0.0141).

The effect of re-expression of *CXCL16* on cell growth was assessed by *in vitro* colony formation assays. Following transfection of a wild-type *CXCL16* expression plasmid (or empty vector) into SKRC39 (an RCC cell line, which is heavily methylated at the CXCL16 promoter), there was a significantly reduced number of G418 resistant colonies (mean 40.1%, *t*=4.16 *P*=0.014) compared to SKRC39 cells transfected with an empty vector control in three independent experiments (Figure 2).

## DISCUSSION

Functional epigenomic screens have proven to be a successful strategy for identifying epigenetically inactivated TSGs in a number of different tumour types (Yamashita *et al*, 2002; Sato *et al*, 2003; Lodygin *et al*, 2005). However, to our knowledge, only one other functional epigenomic study of RCC has been reported. Thus, Ibanez de Caceres *et al* (2006) performed gene expression microarrays in four RCC cell lines (all different to those analysed in our study) after treatment with a demethylating agent (5-AZA-2 deoxycytidine) and a histone deacetylation inhibitor (trichostatin A) and found that between 111 and 170 genes demonstrated a ⩾3-fold upregulation of expression after treatment in each cell line. Then they proceeded to analyse 12 genes that were upregulated ⩾3-fold in at least three of the four cell lines and were expressed in renal tubular cells (*BIRC3*, *NP*, *GADD45A*, *NFKB1A*, *CYCS*, *TGM2*, *IGFBP1*, *COL1A1*, *CTGF*, *IGFBP1*, *GDF15* and *PLAU*) and found that three genes (*IGFBP1*, *IGFBP3* and *COL1A1*) demonstrated tumour-specific methylation and seven genes did not show promoter methylation. Despite differences in experimental details and microarrays employed, their findings are similar to those from our more extensive analysis. Thus, it is clear that although upregulation of gene expression after demethylation treatment will often not indicate promoter region hypermethylation, and even if methylation is present, it may not be tumour specific, this experimental approach does provide a strategy for identifying novel TSGs.

Keratin 19, an intermediate filament protein responsible for the structural integrity of epithelial cells as a gene, frequently demonstrated promoter methylation in sporadic RCC but not in normal kidney tissue from RCC patients and non-cancer patients. Previously, keratin 19 was reported to be downregulated in head and neck cancers. Furthermore, although re-expression of keratin 19 did not affect the growth rate of transfected cell lines, *in vitro* invasiveness after treatment with HGF was reduced after re-expression (Crowe *et al*, 1999).

We found that the transmembrane chemokine CXCL16 (Matloubian *et al*, 2000; Shimaoka *et al*, 2000; Wilbanks *et al*, 2001; Shimaoka *et al*, 2003) was also frequently methylated in RCC cell lines and in primary tumours but not in normal kidney from non-cancer controls. Although *CXCL16* methylation was detected in adjacent normal renal tissue from RCC patients with tumour methylation, this might indicate a premalignant field defect (as described in bronchial epithelium); however, contamination by tumour cells cannot be excluded completely. Interestingly, re-expression of CXCL16 significantly reduced the formation of kidney cell line colonies.

A significant rationale for identifying RCC-associated hypermethylated TSGs is their potential role as biomarkers to identify high-risk individuals and presymptomatic tumours by analysis of urine samples (Battagli *et al*, 2003; Hoque *et al*, 2004). In this context, it is valuable to identify both genes that are methylated early in tumorigenesis (but which may not show tumour-specific methylation) and also genes that are specific for tumours.

The functional epigenomic approach we pursued, in addition to identifying genes associated with tumour-specific methylation, also identified significant numbers of genes that (a) appeared to demonstrate cell line-specific methylation (*SST*, *PTGS1*, *ISG15*, THY1) or (b) were upregulated by demethylation but had no apparent promoter region methylation. The former group, although apparently not frequently methylated in primary tumours, might be considered as candidate genes implicated in progression (assuming that acquisition of methylation in cell culture might have a growth advantage). In this context, it is interesting to note that *PTGS1* methylation is frequent in prostate cancer (Bastian *et al*, 2005), *THY1* has been implicated as a candidate TSG in nasopharyngeal cancer (Lung *et al*, 2005) and *SST* promoter methylation has been described in ∼90% of colorectal cancers (Mori *et al*, 2006).

Genes that were upregulated by demethylation but not methylated included genes that have been described previously as TSGs in other cancer types (e.g. *BAP1* (Jensen and Rauscher, 1999), *IGSF4* (Kikuchi *et al*, 2006), *RRM2* (Gautam and Bepler (2006)), *PMAIP1* (Shibue *et al*, 2003), *claudin-1*; (Higashi *et al*, 2007) and *ICAM1*; (Georgolios *et al*, 2006)). These observations would be consistent with the hypothesis that expression of these genes is reactivated secondary to changes in promoter methylation at upstream regulators. Hence, further analysis of this class of genes may provide insights into key pathways that are dysregulated in RCC and identify candidate upstream regulators for epigenetic analysis.

**Figure 3:**
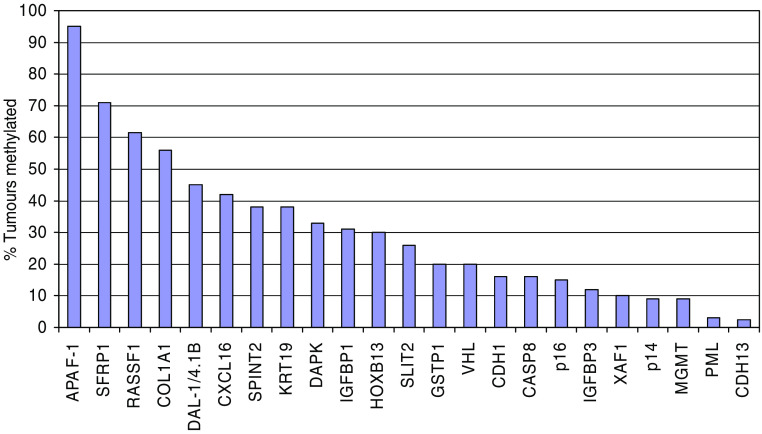
Frequency of gene promoter methylation in RCC. Data were derived from the present study and (Merlo *et al*, 1995; Dreijerink *et al*, 2001; Morrissey *et al*, 2001; Yoon *et al*, 2001; Morris *et al*, 2003; Gonzalgo *et al*, 2004; Morris *et al*, 2005; Christoph *et al*, 2006; Okuda *et al*, 2006; Yamada *et al*, 2006; Dahl *et al*, 2007; Gumz *et al*, 2007; Kempkensteffen *et al*, 2007). Abbreviations: *APAF-1*, apoptotic protease activating factor; *CASP8*, caspase 8; *CDH1*, cadherin 1; *CDH3*, cadherin 3; *COL1A1*, collagen type I, alpha-1; *CXCL16*, chemokine, cxc motif, ligand 16; *DAL-1/4.1B*, differentially expressed in adenocarcinoma of the lung/nonerythroid protein 4.1, brain type 4.1b; *DAPK*, death-associated protein kinase 1; *GSTP1*, glutathione-*S*-transferase, PI; *HOXB13*, homoeobox B13; *IGFBP1*, insulin-like growth factor-binding protein 1; *IGFBP3*, insulin-like growth factor-binding protein 3; *KRT19*, keratin 19; *MGMT*, methylguanine-DNA methyltransferase; *p14*, cyclin-dependent kinase inhibitor 2a alternative reading frame; *p16*, cyclin-dependent kinase inhibitor 2a; *PML*, acute promyelocytic leukaemia (inducer of); *RASSF1*, Ras association domain family protein 1; *SFRP1*, secreted frizzled-related protein 1; *SLIT2*, slit, *Drosophila*, homologue of 2; *SPINT2*, serine protease inhibitor, Kunitz-type 2; *VHL*, Von Hippel–Lindau syndrome gene; *XAF1*, XIAP-associated factor.

We have reported the identification and systematic analysis of candidate RCC TSGs identified from a functional epigenomics screen. To date, the only two functional epigenomic studies performed in RCC have each analysed four RCC cell lines on arrays containing <15 000 genes, but each have identified novel genes with tumour-specific methylation. Compared to other genes reported to be methylated in RCC, *SPINT2*, *CXCL16* and *KTN19* are relatively frequently methylated (Figure 3). Therefore, we suggest that further, more extensive, studies are warranted to identify additional potential RCC TSGs and methylated biomarkers for early cancer detection.

## Figures and Tables

**Table 1:** Genes differentially expressed and frequently methylated in cell lines and/or tumours

**Gene ID**	**Chromosome position**	**Gene name**	**Gene symbol**	**Function**	**Silenced in cell line**	**Promoter methylation in cell line**	**Promoter methylation in T**	**Promoter methylation in N**	**Promoter methylation in NDN**
AA458849	19q13.2	*Serine peptidase inhibitor, Kunitz type 2*	*SPINT2*	Serine protease inhibitor	5/11	4/9	45/118 (CCRCC: 22/74, Pap: 20/44)	2/38	0/6
AA464250	17q21.2	*Keratin 19*	*KTN19*	Protein binding, structural	5/11	4/10	25/66 (CCRCC: 20/51, Pap: 5/15)	3/22	0/6
AA411656	17p13.2	*Chemokine (C-X-C motif) ligand 16*	*CXCL16*	Chemokine activity	4/11	5/8	26/62 (CCRCC: 20/47, Pap: 6/15)	9/21	0/6
AA496283	11q23.3	*Thy-1 cell surface antigen*	*Thy1*	Integrin binding, Rho GTPase activator	5/11	6/7	0/20	0/15	0/6
AA446108	9q34.11	*Endoglin (Osler–Rendu– Weber syndrome 1)*	*ENG*	Protein binding	5/9	4/9	8/9	16/21	6/6
R51912	3q27.3	*Somatostatin*	*SST*	Hormone activity	5/9	8/8	0/20	0/10	0/6
AA45668	9q33.2	*Prostaglandin-endoperoxide synthase 1*	*PTGS1*	Peroxidase activity	6/11	6/9	0/20	0/10	0/6
AA406020	1p36.33	*ISG15 ubiquitin-like modifier*	*ISG15*	Protein binding	8/9	6/9	0/20	0/15	0/6

Abbreviations: CCRCC=clear cell renal cell carcinoma; N=adjacent normal tissue; NDN=non-disease-normal tissue, kidney tissue obtained from non-cancerous kidneys; Pap=renal papillary tumour; T=sporadic RCC tumour.

Expression analysis was carried out by semiquantitative RT–PCR. All promoter methylation analysis was performed by sequencing of bisulphate-modified DNA.
