# Supplementary material for: Functional epigenomics approach to identify methylated candidate tumour suppressor genes in renal cell carcinoma
Source: Br J Cancer. 2008 Jan 15;98(2):496–501. doi: 10.1038/sj.bjc.6604180 (PMC2361461; doi:10.1038/sj.bjc.6604180)
Supplement: Supplementary Table 1 [file 6604180x1.doc]

Supplementary Information

BISULPHITE SEQUENCING PRIMERS

| KTN19 | F: GGA GTT TGT AAA TTT TTT AGG GTT TAG ATA T |
| --- | --- |
|  | R: CAT AAT TAA CTT CTC RTT ACC CRC CAA |
|  | IF: TAG TTT TTG AGA TTA GGG TTG TTT YTG TYG TGT |
| BAP1 | F: GTY GGA GAA GGG GTT TAA GGA GTA TTT |
|  | R: CAA TAC TAA ACC CRA ATA CTA CRA AAA AAA |
|  | IF: GTT GTA TTG GAG TTT TAG TYG TTG ATA TAG G |
|  | IR: CCC RAA AAT AAC TAC CAC RACCTA TAT C |
| RRM2B | F: GGA GGT GGG TTG AGG AGT TTA GTT TTT TTT |
|  | R: CTT ATC ACC ATC CCA AAT AAT ATA CAC CT |
|  | IR; ATT TCC CTA ACC AAA ACT CCA AAA CAA AAA |
| ICAM | F: TTY GTG TTA GTT AGG TGT GGA YGT GAT T |
|  | R: CCA TAA CRA AAC TAA AAT TAC AAC TCT TAA CAA |
|  | IF: GAG TTT TAG TTT ATY GTT TTG TGA AAT GGA TAT |
| CLAUDIN1 | F: GTG TTA GGA TTT ATA AGT ATA GTG TAT AG |
|  | R: CAC TCA CTA CTC AAA TTC AAC AAA AAA TC |
|  | IR: CAA AAA ACR AAA AAT TTA CAT ATA AAC AAC C |
| PHD3 | F: GGT TGG YGT YGG GTT TTT TGG GAT TTT T |
|  | R: CTA ATA ACC TCR CAA CCC TCC TCR TTA CCC |
|  | IF: GGT AGT TGT AGG TTT TTG AAT TTY GGG |
|  | IR: CCC CAA CRT ACA ACC AAA AAA AAA ACR ATC |
| MUC1 | F: GGT TAG GTT GGT TTT GAA TTT TTG ATT |
|  | R: ATT CCC TCC TTA AAA AAA ACC AAA AAA AAA ATA |
|  | IR: AAA TCC TTC CTT ACC ACT CTC CAC CAC TAA |
| PMAIP1 | F: TTT AGY GTY GGT GGA TAG GAA GTT AGG GTT |
|  | R: CAA AAC RAA ACC RAA CCR AAT ACR CAT CCT AAA A |
|  | IF: GAA GTT AGG GTT TTT GTA GGG AGG YGT TYG TTT |
| SST | F: TTT TGY GAG GTT AAT GGT GYG TAA AAG GGT |
|  | IF: TTG GGG GYG TTT TTT AGT TTG AYG TT |
|  | R: CCR AAA AAC RTC RAA AAA ATC TCC TTA CCT A |
| EHM2 | F: AGG GAG GAG GGY GTG TTG TAG TTT |
|  | R: CCR ACR CAA AAA CCR CAA CAT CCT AAC TAA |
|  | IF: GAG TGG GAG YGA GYG YGT TAG GAT T |
| IGSF4 | F: GGA TAG AGG ATT TTT TTA AGG GAG ATT TTT T |
|  | R: ATA CCC ACA CCT ACC TAT AAA AAT CAA TA |
|  | IF: GTA TAA GAA GTT AGA TTY GTT TTT TGG AGT T |
| ISG15 | F: TAG TTA GTG TTT TGT GTG TGG TGG GTT |
|  | R: AAT CAA CCA AAA CAA ATR ATC CTA CAC ACC CT |
|  | IF: TTG GTG AGG AAT AAT AAG GGT YGT AGT AGT ATT |
| ENDOGLIN | F; GGT TGG ATG AGT TAG GAG TTT TTT GTT GT |
|  | R: CAC CCT AAA TCC CTA AAC ACC TAC TTA TA |
|  | IF: GGA TTG TTG TTG TTA TTG TTA TTT ATT GGA GTT TAG |
| THY1 | F: TAG GTT TGT TAG TTA TAG GGT GAG GGT TTT |
|  | R: AAC TAT CCT TAA TAC CCT TCC TCC CTA |
|  | IF: TTT YGT TTT TGA TTG GTT GAG TTT TYG GTT TT |
| CXCL16 | F: TTA GAG AGG AGA GAG GGT TAA TTT TAT T |
|  | R: ACC CRA AAA ACC RAT TCR ATT CRA TTC AAA |
|  | IF: TTG YGT GGY GGG TAG TTT YGG TAG ATT TAG TT |
| SLC25A21 | F: TTG GTT TGT TAG GAG GTG TAA GTG TYG TTT |
|  | IF: GTT TTA GGT TTG GGT TAT TTT ATT TGA GTT ATT |
|  | R: ACC TAC TAA TCC AAA AAA CCC CRA CTA AAC TTT |
| PTGS1 | F: GTA GGG TTT TTA ATA GAG YGT TTY GGT TTG ATT |
|  | R: CT TCC CAC CTT ACA CCT TCT TAA CAA |
|  | IR: CCR AAA ATC TCR CCT TAC CCR AAA TAC TAA |
| MYL2 | F: ATA TTT TAT ATG GTT TAT AGG GAG TTA TAG AG |
|  | R: ACC CTA AAC CRT TAA ACC TAT CCT CAA AA |
|  | IF: GAA GTT GTA TTT TTA YGG TTT ATA TAA GTG G |
| H2B | F: GGA YGY GGA AAG TAG GGA GGT AAG GTT |
|  | R: ACC TAA ATA TTA AAC AAA ACR CCR CCC TAA |
|  | IF: TTG GTT TTT AGT TTT YGG TGG GGY GAG TGT |
| ECE1 | F: GAT TTA AGA TTT TTT TGA AGG AGG GGT TAT |
|  | R: ACA ACA CRA ACT CCC TCA ACR CCA TAA |
|  | IF: TTT TTT TGT TTG TTA GGG GTT TTA GTT TGG |
| CTGF | F: GTT AGG ATT AAT TYG GTG TGA GTT GAT GAG GT |
|  | IF: GGA ATG YGA GGA ATG TTT TTG TTT GTG TAG GAT |
|  | R: CRA AAC CCA TAC TAA CRA CRA TCA TAA TTA ACA CTA |
| GPR39 | F: TTT TGT TAG AGT TTT TAG TTT TTT TTG TTG TTT |
|  | R: AAA ACC ATA AAA AAA AAC ACC AAA CTA CCA AA |
|  | IF: TAG GGA TYG TGT TTA TGT GTT GAG TTA AAT |
| SEMA5A | F: GAT TTA GTG AGA YGG GGG AGG GAG TT |
|  | R: CCR ACR CCR CCT TCC CRA CAA ATA AAT CRA |
|  | IF: TTY GTA TTG GGG AGG TTG GAA TGG AAT |
| FBLN1 | F: GGT GGG GGA ATC YGG TTT TGA AAT AAG TTT |
|  | R: CTC CRC CRC RCC CTC CTC CCR TT |
|  | IF: TTT TTA GGT YGG GGA GGA GAT GAG GAT |
